# Axial Length as a Risk Factor for Idiopathic Macular Hole: A Five‐Year Prospective Study

**DOI:** 10.1155/joph/5539015

**Published:** 2026-04-10

**Authors:** Yongwun Cho, Hayoung Byun, Woong-Sun Yoo, Inyoung Chung

**Affiliations:** ^1^ Department of Ophthalmology, Gyeongsang National University Hospital, College of Medicine, Gyeongsang National University, Jinju, South Korea, medcol.mw; ^2^ Department of Rehabilitation Medicine, Gyeongsang National University Hospital, College of Medicine, Gyeongsang National University, Jinju, South Korea, medcol.mw; ^3^ Health Science Institute, Gyeongsang National University, Jinju, South Korea, gnu.ac.kr

**Keywords:** axial length, fellow eye, macular hole, prospective study

## Abstract

This 5‐year prospective follow‐up study investigated whether short axial length influences the incidence of new idiopathic macular holes in fellow eyes of patients with unilateral macular hole. A total of 100 unilateral idiopathic macular hole patients and age‐ and sex‐matched controls were followed for 5 years. The incidence of macular hole in the fellow eye was prospectively observed, and the mean axial length was compared between fellow eyes that developed new macular holes and those that did not. During the follow‐up, 11 of 100 fellow eyes in unilateral macular hole patients developed a new macular hole, whereas no cases occurred in the control group. The mean axial length of fellow eyes with new macular hole was 21.42 ± 0.39 mm, significantly shorter than that of fellow eyes without new macular hole, which measured 22.98 ± 0.78 mm (*p* < 0.001). These findings demonstrate that short axial length is significantly associated with the development of new idiopathic macular holes in fellow eyes and provide prospective evidence supporting short axial length as a risk factor. Given its accessibility and practicality in clinical practice, axial length may serve as a useful parameter for identifying high‐risk patients and guiding follow‐up strategies.

## 1. Introduction

Many hypotheses have been proposed regarding the mechanism by which idiopathic macular holes occur, but it has not yet been clearly elucidated. Recently, tangential traction in the anteroposterior direction of the vitreous due to contraction of the perifoveal posterior vitreous cortex has been explained as one of the important mechanisms in the development of idiopathic macular holes [1–6]. There are various hypotheses regarding the causative factors of macular hole, such as age, gender, and myopia, but there is still no clear conclusion [1, 6, 7]. Previous studies have reported that axial length is a causal factor for macular holes, but conflicting opinions have been presented. In addition, there are various hypotheses about the mechanism by which short or long axial length causes macular holes, but there have been no established theories or results yet [8–11].

After performing macular hole surgery for many years, the authors recognized that patients with idiopathic macular hole tend to have a short axial length. Therefore, the relationship between axial length and the occurrence of macular holes was investigated through a previous retrospective study. As a result, it was confirmed that patients with unilateral macular hole had a shorter axial length when compared to a randomly selected control group. In addition, it was found that the fellow eye without a macular hole also had a shorter axial length compared to the control group because the axial length was similar in both eyes of a person [1]. The authors hypothesized that if short axial length affects the occurrence of the macular holes, the incidence of macular holes in the healthy fellow eyes of unilateral macular hole patients would be higher than that of the control group. We designed a prospective 5‐year follow‐up study to confirm this hypothesis.

## 2. Methods

Based on our previous retrospective case–control study that analyzed patients diagnosed with macular holes at our hospital from January 2015 to December 2020, we designed this study as a 5‐year prospective follow‐up to evaluate the incidence of new macular holes in fellow eyes. The results of optical coherence tomography (OCT) were used for the Gass classification, and patients diagnosed with full‐thickness macular hole among Stage 2 to 4 in one eye were included [2]. Secondary macular holes such as macular holes occurring after trauma, surgery, or high myopia with posterior staphyloma were excluded, and cases with an ophthalmologic history or surgical history other than macular holes were excluded from the study. For the control group, simple cataract patients treated at our hospital during the same period were included. Secondary cataracts such as trauma, uveitis, and high myopia including posterior staphyloma were excluded, and cases with an ophthalmologic history or surgical history other than simple cataract were also excluded from the study. The control group was randomly selected by matching the age and gender of macular hole patients using the precataract surgery examination data within the study period. Additionally, we collected information from 100 eyes of 100 patients, including only the single‐eye data for which surgery was planned for each patient, for the diversity of control groups.

Divided into a group with an eye with a macular hole, a healthy fellow eye without a macular hole, and a control group, demographic data such as age and gender were collected. The refractive power and anterior chamber depth were collected and analyzed through medical records. Refractive power was calculated with spherical equivalent. The axial length was measured using IOL master (Carl Zeiss, Jena, Germany). For this prospective follow‐up study, we observed the healthy fellow eyes of unilateral macular hole patients and the control group for 5 years. During the follow‐up period, patients who developed full‐thickness macular holes were investigated using the same criteria as before. Patients were excluded from the study if new ophthalmic diseases or surgical history, other than cataract surgery or macular hole, occurred during follow‐up. Statistical tests used independent‐sample *t*‐test and Mann–Whitney *U* test. All statistical analyses were performed using SPSS (Version 21.0, software for Windows; SPSS Inc., Chicago, IL, USA), and a *p* value of less than 0.05 was considered statistically significant. This study was approved by the Institutional Review Board of Gyeongsang National University Hospital (IRB No. 2021‐01‐017), and the requirement for informed consent was waived due to the retrospective and observational nature of the research. All procedures adhered to the tenets of the Declaration of Helsinki.

## 3. Results

Demographic data and the comparison of mean axial length in three eye groups followed the previous study [1]. There were 15 men and 85 women in both the macular hole and control groups. The mean age of the macular hole group and control group was 61.3 ± 3.76 and 62.1 ± 1.32 years old, respectively. There were no statistically significant differences in age and gender between two groups [1]. In the macular hole group, the mean anterior chamber depth on the eye with a macular hole and healthy eye was 2.62 ± 0.21 and 2.63 ± 0.21 mm, respectively. The mean anterior chamber depth in the control group was 2.67 ± 0.19 mm. There was no significant difference in comparison between the eye with a macular hole and the healthy fellow eye, the eye with macular hole and the control group, and the healthy eye and the control group [1]. The mean refractive power was 0.86 ± 1.10 D, 0.82 ± 1.11 D, and 0.56 ± 1.25 D in eyes with a macular hole, healthy fellow eye, and eyes of control group, respectively. There was no statistical difference between the refractive powers of three groups [1]. In the macular hole group, the mean axial length of the macular hole eyes was 22.71 ± 0.92 mm, and the average axial length of the healthy fellow eyes was 22.81 ± 0.89 mm. Although eyes with a macular hole were shorter than the healthy eyes, there was no statistical significance. The mean axial length of the control group was 23.37 ± 0.68 mm. The mean axial length of macular hole eyes was statistically significantly shorter than that of the control group, and a significant difference was also found when comparing the healthy fellow eyes of the macular hole group with eyes of the control group (*p* < 0.001, Table 1) [1].

**TABLE 1:** The mean axial length and occurrence of new macular hole in eyes with macular hole, fellow eyes of patients, and the control group.

	**Macular holes (*n* = 100)**	**Fellow eyes (*n* = 100)**	**Control (*n* = 100)**	**p**value**^∗^**	**p**value**^∗∗^**	**p**value**^∗∗∗^**

Axial length (mm)	22.71 ± 0.92	22.81 ± 0.89	23.37 ± 0.68	0.477	< 0.001	< 0.001
New macular hole occurred (*n*)		11	0			

*Note:* Values are presented as mean ± SD.

^∗^Compared macular holes and fellow eyes.

^∗∗^Compared macular holes and control.

^∗∗∗^Compared fellow eyes and control.

**FIGURE 1:**
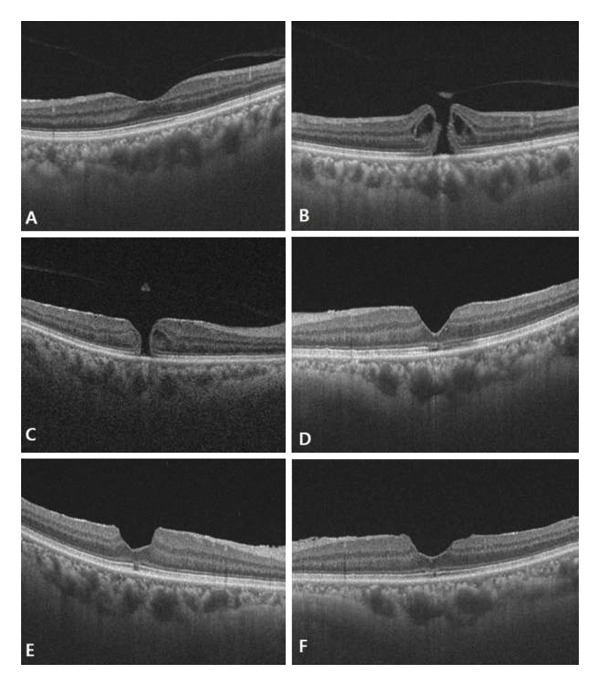
Representative serial OCT images of a single patient during long‐term follow‐up. (A, B) Baseline images of the right (A) and left (B) eyes. (C, D) Images at the same follow‐up time point showing macular hole development in the right (C) eye and postoperative status of the left (D) eye. (E, F) OCT images obtained at the same postoperative time point, showing the postoperative status of the right eye one year after surgery (E) and the left eye two years after surgery (F).

During the 5‐year prospective follow‐up period, no macular holes developed in the control group, whereas 11 fellow eyes in the macular hole group developed new macular holes (Table 1). Among the 11 patients who developed a macular hole in the fellow eye during follow‐up, 4 were male and 7 were female. There were no eyes that underwent additional surgery other than cataracts or had any other ocular diseases other than macular holes. The mean axial length of the 11 fellow eyes with macular holes was 21.42 ± 0.39 mm, which was significantly shorter than the mean of 22.98 ± 0.78 mm among the remaining 89 fellow eyes without macular holes (Table 2). A representative example of a patient with short axial length who developed a macular hole in the fellow eye during follow‐up is presented (Figure 1).

**TABLE 2:** Comparison of the mean axial length in cases with and without new macular hole among fellow eyes of patients with unilateral macular hole.

	**Macular hole (*n* = 11)**	**No macular hole (*n* = 89)**	**p** **value**

Axial length (mm)	21.42 ± 0.39	22.98 ± 0.78	< 0.001

*Note:* Values are presented as mean ± SD.

## 4. Discussion

Since the mechanisms of macular hole formation involving tangential and anteroposterior vitreous traction were first proposed in 1995 [3], multiple hypotheses have subsequently been suggested. Axial length may influence these tractional forces, and our previous retrospective study demonstrated that macular hole patients had shorter axial length than control [1]. In this prospective 5‐year follow‐up study, we examined patients with unilateral idiopathic macular hole and found that new macular holes developed in 11 fellow eyes of these patients, whereas no new macular hole occurred in the control group. We also found that the mean axial length of fellow eyes that developed new macular holes was significantly shorter than that of fellow eyes without new macular holes.

A previous study reported that the normal axial length by age group was reported to be 23.40 mm for those between 60 and 69 years, which is similar to the axial length of the authors’ control group [12]. In addition, it is considered appropriate as a control group because the axial length of the control group in this study showed similar result to the previously published study on the relationship between macular hole and axial length [9, 10]. Also, the macular hole group in this study showed no statistical differences from the control group in terms of gender, age, refractive power, and anterior chamber depth. Additionally, patients with cataract surgery or other ophthalmological diseases were excluded. Therefore, we believed that appropriate control group was selected, and appropriate data were collected to investigate the incidence of an idiopathic macular hole. Because age and sex were matched at baseline, the observed association between axial length and macular hole development is unlikely to be explained by these factors.

Several previous studies have investigated the relationship between axial length and idiopathic macular hole, with conflicting results. A previous report [7] described a higher incidence of macular holes in younger patients with severe myopia, although refractive power did not influence surgical outcomes. A previous study [9] found significantly shorter axial length in macular hole patients compared with controls, which is consistent with our findings, while another report [8] showed shorter axial length in affected eyes compared with fellow eyes. In contrast, two studies [10, 11] reported longer axial length in macular hole patients. These discrepancies may be related to differences in study design, patient selection, and control groups. Nonetheless, our results add prospective evidence that short axial length is associated with increased risk of macular holes, aligning with several previous reports while addressing limitations of retrospective designs.

In recent studies on macular holes, tangential traction forces on the retinal surface around the fovea and optic nerve were known to have great influences on the occurrence of macular holes [2–6]. Additionally, it was known that incomplete posterior vitreous detachment could increase the tangential traction force on the retinal surface [13]. It has been hypothesized that in patients with macular holes, short axial length created a small vitreous volume, which affected incomplete posterior vitreous detachment and caused macular holes [9]. In other study that postmortem pathological analysis of the eyes of patients with idiopathic macular hole, it was found that when friction and deformation of the eye were chronically applied with small forces, the force was transmitted to the incompletely liquefied vitreous cortex. It is said that this force was transmitted from the macular hole to the posterior vitreous cortex near the narrowed fovea, creating a strong tangential traction force and causing a macular hole [14].

Analyzing the authors’ research results citing previous studies, a short axial length would create a small volume in the vitreous cavity. As a result, it is thought that the force generated from friction and deformation of the eyelid and eyeball that may occur in daily life would reach the retina surface where tangential traction occurs more quickly and often than the long axial length. Although this phenomenon would be a small force, if it continues for a long time until the average age of onset of macular holes, it may affect the occurrence of macular holes more than in long axial lengths by affecting the tangential traction force of the retinal surface.

In a previous study comparing both eyes of macular hole patients, the eye with a macular hole had a shorter axial length than the healthy fellow eye [8]. In our study, the lesion side in the macular hole group showed a shorter axial length than the healthy eye, without statistical significance. The axial length was shorter in eye with macular hole and fellow healthy eye than that in the control group. The axial length is generally known to be similar in both eyes, so there might be no difference between two eyes of macular hole patients and is considered to be evidence confirming that the axial length values of both eyes were appropriately measured.

In this study, we hypothesized that if the axial length of both eyes was measured similarly and the short axial length was the cause of the macular hole, the occurrence of macular holes would be more likely if follow‐up was performed in the healthy eyes of patients with unilateral macular holes [1]. Also, it was thought that the relationship between axial length and macular hole could be known by comparing the axial length of eyes with and without new macular holes. Therefore, the authors had a follow‐up period of 5 years. Comparing our results with previous studies, the present study provides strong evidence that short axial length influences the occurrence of macular holes because macular holes developed in 11 fellow eyes of patients with a unilateral macular hole during the 5‐year prospective follow‐up, while no macular holes occurred in the control group. In addition, the axial length of these 11 fellow eyes that developed macular holes was significantly shorter than that of fellow eyes without macular hole occurrence. These findings strengthen the hypothesis that short axial length, as previously claimed by the authors, contributes to the occurrence of macular holes.

This study was conducted on 100 patients, and although more analysis was performed than previous publications, it has the disadvantage of having a limited number of patients. Also, the authors’ study is limited to idiopathic macular holes, and the mechanism of occurrence might be different from high myopic macular holes and macular holes that occur after vitrectomy. Therefore, short axial length might be a limited risk factor for idiopathic macular holes, and future study into macular holes caused by other mechanisms is needed.

To date, various hypotheses have been proposed regarding the causes of macular holes. In addition, some papers contradicted the previous study’s claim that long axial length has an effect, so further investigation was needed. Through this study, the authors believe that the hypothesis that short axial length can affect the occurrence of macular holes has become more credible. To our knowledge, this is the first prospective 5‐year follow‐up study to directly evaluate axial length as a risk factor for the incidence of idiopathic macular holes in fellow eyes. Although only 11 new cases were observed, these represent prospectively documented incident events rather than retrospective inferences, thereby strengthening the validity of the findings. Notably, the association between short axial length and macular hole risk persisted despite the modest number of events, supporting the role of axial length as a practical and clinically meaningful marker for risk stratification.

While this study was limited by its single‐center design and relatively small sample size, the prospective nature and 5‐year follow‐up confer unique value to the existing literature. Larger multicenter studies will be essential to validate these findings and to determine whether axial length can be integrated into predictive models for macular hole occurrence.

## 5. Conclusion

This 5‐year prospective follow‐up study demonstrates that short axial length is associated with a significantly increased risk of idiopathic macular hole development in the fellow eye of unilateral macular hole patients. Because axial length is routinely measured and easily obtainable in clinical practice, it may serve as a practical parameter for identifying high‐risk patients and guiding follow‐up intervals. Regular OCT surveillance should be considered for patients with short axial length in the fellow eye.

## Author Contributions

All authors contributed to the study conception and design. Material preparation and data collection and analysis were performed by Yongwun Cho, Woong‐Sun Yoo, and Inyoung Chung. The first draft of the manuscript was written by Yongwun Cho and Hayoung Byun. All authors commented on previous versions of the manuscript.

## Funding

The authors declare that no funds, grants, or other support were received during the preparation of this manuscript.

## Disclosure

All authors read and approved the final manuscript.

## Ethics Statement

This retrospective and prospective observational study was approved by the Institutional Review Board (IRB) of Gyeongsang National University Jinju Hospital (IRB No. 2021‐01‐017). The need for informed consent was waived because of deidentified data and the study adhered to the tenets of the Declaration of Helsinki.

## Conflicts of Interest

The authors declare no conflicts of interest.

## Data Availability

The data that support the findings of this study are available on request from the corresponding author. The data are not publicly available due to privacy or ethical restrictions.
